# Editorial: Neuromodulatory ascending systems: Their influence at the microscopic and macroscopic levels

**DOI:** 10.3389/fncir.2022.1028154

**Published:** 2022-10-26

**Authors:** Giuditta Gambino, Rebecca Bhik-Ghanie, Giuseppe Giglia, M. Victoria Puig, Juan Ramirez-Villegas, Daniel Zaldivar

**Affiliations:** ^1^Department of Biomedicine, Neurosciences and Advanced Diagnostics, University of Palermo, Palermo, Italy; ^2^National Institute of Mental Health (NIH), Bethesda, MD, United States; ^3^Catalan Institute of Nanoscience and Nanotechnology (IN2), Universitat Autònoma de Barcelona (UAB), Barcelona, Spain; ^4^Institute of Science and Technology Austria, Klosterneuburg, Austria

**Keywords:** neuromodulation, networks, neuroanatomy, circuits, brain disorders

Brain activity and behavior are constantly changing (Puig et al., 2014; Disney, 2021). Recent studies in both animal models and humans have revealed that such variations are not random in nature but controlled through slow-acting neuromodulatory systems which influence our ability to process the influx of information, make decisions and take appropriate actions. Traditional anatomical, electrophysiological, stimulation, and lesion studies have revealed several important groups of pathways which are typically localized in brainstem/hypothalamic centers (McCormick et al., 2020). These so-called neuromodulatory ascending systems are known as the basal forebrain cholinergic system (ACh), noradrenergic brain stem, serotonergic brainstem, dopaminergic system (DA), endocannabinoids, oxytocin, nitric oxide-, and others less investigated (Puig and Gulledge, 2011; Gambino et al., 2020; Tong et al., 2021). All of these systems are known to broadly project to different brain areas, affect interneuronal communication, change synaptic efficacy, and influence cognitive processes.

Hence, to understand the functioning of a given brain network, it is not only sufficient to know its elements, but one needs to understand how these elements are interlinked and dynamically interact (Zaldivar et al., 2022). In recent years, great progress has been made towards characterizing the role of neuromodulation and it is, therefore, more than timely that Frontiers Research Topic has chosen to devote a special topic on this subject. This brief editorial attempts to provide an overview about the publications in this special issue and should serve as an introduction to those unfamiliar with the topic.

Neurotransmitters and neuromodulators often share similar molecular elements, with main differences depending on the structure and function of their respective receptors. Neurotransmitters are released by presynaptic neurons, they directly and immediately influence their postsynaptic targets. The fast influence of many neurotransmitters comes about because their receptors are harnessed to ion-channels that open almost instantaneously in the presence of the neurotransmitter ligand to modulate ion conductance. Neuromodulators tend to act much more slowly and endure for a longer duration, since their receptors are coupled to cell physiology *via* second messenger pathways. Ultimately, the influence of neuromodulation tightly depends on receptor location, distribution and density. Indeed, these aspects were essential in the study of Ogata et al., where authors evaluated how the uneven balance of dopamine receptors affects the role of medium spiny neurons in the direct and indirect motor pathways (Albin et al., 1989; Kravitz et al., 2010). This prompts speculation that axons of these two pathways might independently coordinate their responses to shape motor- or limbic-related information processing in the striatum.

The cholinergic system is one of the most widely investigated neuromodulatory systems as it is involved in multiple aspects of sensory processing (Runfeldt et al., 2014; Minces et al., 2017; Zaldivar et al., 2018), learning and memory (Atherton et al., 2015; Ramirez-Villegas et al., 2021), and it has been causally linked to different types of dementia (Baxter and Chiba, 1999). However, to what extent does the role of ACh result from the local influence on neural circuits (Zaldivar et al., 2018) or from the interactions between multiple and remote circuits instantiated by ACh itself (Runfeldt et al., 2014)? In the attempt to infer this differential role of cholinergic modulation, Tsolias and Medalla found that two areas of the prefrontal cortex—commonly associated with arousal and motivation during executive functions—have distinct regional and laminar receptor distribution and density. This clear difference in ratio of muscarinic receptors type 1 and 2 in these areas is surprising since both regions were assumed to share similarities in their anatomy and function. It is not far-fetched to hypothesize that these findings suggest that cholinergic neuromodulatory control might optimize the bottom-up and top-down signals at different processing stages to facilitate the transfer of information and during similar time scales.

Most neural circuits in the animal kingdom must be sufficiently flexible to allow behavioral adaptation in response to their unique ecological and social landscape. Neuromodulators lead to the assemblage of information between neural circuits so that they can be orchestrated to support behaviors in the species' repertoires. However, such flexibility is not only given by the diversity of neuromodulators, neurons and receptors, but it is also attained by the neuron's capability to be influenced by more than one neuromodulator (Marder et al., 2014). This is clearly highlighted by the findings from Kotsyuba and Dyachuk, who found that in some crustaceans the synthesis of dopamine, serotonin and acetylcholine takes place in the optic nerve. Markedly, authors identified that axons expressing enzymes for the synthesis of these neuromodulators were close to the neurosecretory cells which can further influence animals' behavior.

Understanding how disturbances in neuromodulatory pathways might yield to brain disorders is of high importance for neuroscience research. Although we have learned a great deal of information about the anatomical abnormalities associated with brain disorders we are still far from understanding how these abnormalities may be causally linked. Zhao et al. provided an in depth review about the Autism Spectrum Disorder (ASD) and the role that disturbances in neuromodulatory ascending pathways might play in the development of ASD. Zhao et al. proposed that other less investigated neuromodulatory systems might enhance our understanding about the origin, progression, and treatment of this disorder. Authors emphasized in animal models and in clinical studies using treatments targeting the oxytocinergic and serotoninergic systems have shown to exert beneficial influence (Wichers et al., 2019).

The key problems in the field of neuromodulation are not different from critical questions that have long been posed by the systems neuroscience community. Among others, how does neuromodulatory ascending pathway inputs provide neurons with the flexibility to adjust neural activity? How do neuromodulators orchestrate the activity to give rise to the large repertoire of behaviors in the animal kingdom? To succeed in this endeavor, we need a combination of technological advances, a better understanding of anatomy and physiology and well-crafted experimental designs. In accordance with these questions, the present research topic has contributed to determining the multifaceted role of ascending neuromodulatory pathways in neural networks and behavioral dynamics from basic to more complex experimental models.

## Author contributions

All authors listed have made a substantial, direct, and intellectual contribution to the work and approved it for publication.

## Funding

This work was supported by a DFG grant ZA990/1 to DZ.

## Conflict of interest

The authors declare that the research was conducted in the absence of any commercial or financial relationships that could be construed as a potential conflict of interest.

## Publisher's note

All claims expressed in this article are solely those of the authors and do not necessarily represent those of their affiliated organizations, or those of the publisher, the editors and the reviewers. Any product that may be evaluated in this article, or claim that may be made by its manufacturer, is not guaranteed or endorsed by the publisher.
